# The benefits of inositol-stabilized arginine silicate as a workout ingredient

**DOI:** 10.1186/1550-2783-12-S1-P14

**Published:** 2015-09-21

**Authors:** S Rood-Ojalvo, D Sandler, E Veledar, J Komorowski

**Affiliations:** 1 Nutrition 21, LLC, Purchase, Harrison, NY, USA; 2 StrengthPro Inc, Golden, CO, USA; 3 Emory University, Atlanta, GA, USA

## Background

The purpose of this study was to examine the benefits of inositol-stabilized arginine silicate (ASI; Nitrosigine^®^) as a workout ingredient in healthy adults. ASI has been previously shown to significantly enhance blood levels of arginine up to six hours post-dose and increase nitric oxide levels. To investigate reports of enhanced energy, increased muscle pump and stamina during workouts, and faster muscle recovery post-workout, ASI (1,500mg/day) was tested in a double-blind placebo-controlled crossover-design (DBPC-X) study using the POMS vigor-activity and fatigue-inertia sub-scores, blood flow measurements, leg circumference measurements, and biomarkers of muscle recovery (creatine kinase (CK) and lactate dehydrogenase (LDH)) as outcome measures.

## Methods

The DBPC-X study was conducted in male subjects (N = 16 per group) who had limited exercise routines prior to participating in the study. These subjects took ASI daily for 4 days. Subjects had baseline measurements drawn at the hour 0 visit, took the study product, and completed an intense leg extension exercise protocol to induce muscle soreness. Subjects returned after 24, 48, and 72 hours for additional study measurements. After 72 hours, subjects repeated the leg extension exercise protocol. There was a seven-day washout between test products. Between-product assessments were primary endpoints and within-product assessments secondary endpoints.

## Results

Sixteen (16) healthy male subjects (19-33 years of age) completed the study. Perceived energy, measured using the POMS vigor-activity sub-scores, significantly increased after 72 hours compared to placebo (p = 0.039). At 72 hours, perceived fatigue, measured using the POMS fatigue-inertia sub-scores, significantly decreased in the ASI group (p = 0.041) from pre-dose, compared to a non-significant change in the placebo group (p = 0.580); p = 0.055 between groups.

Hyperemia, measured using leg circumference, increased significantly in the ASI group by 1.8cm (p = 0.001) at 72 hours from pre-dose, compared to a non-significant increase in the placebo group by 0.8cm (p = 0.091); p = 0.070 between groups.

Blood flow, measured by blood velocity through the femoral artery using a Doppler Ultrasound, increased 59.9 cm/s in the ASI group (p < 0.005) and 49.9cm/s in the placebo group (p < 0.005) after exercise on Day 3; p = 0.2 between groups.

CK levels significantly decreased in the ASI group at 24 (p = 0.040), 48 (p = 0.017) and 72 (p = 0.034) hours post-exercise compared to the placebo group. Immediately post-exercise at the hour 0 visit, ASI supplementation led to 44% less muscle damage, measured by CK levels, than placebo (p = 0.057). LDH levels significantly increased from baseline immediately after exercise in the placebo group (p = 0.015), but not in the ASI group (p = 0.366); p = 0.133 between groups. No safety concerns were raised by this study.

## Conclusion

Both primary and secondary endpoints show that daily doses of ASI prior to workout significantly increased pre-workout energy levels, increased muscle pump immediately following a workout, and decreased biomarkers of muscle damage immediately after a workout and during recovery. These results demonstrate multiple benefits of ASI as a functional workout ingredient.

